# Being Wardable: Patient, Family and Staff Member Views of the Otherness of Critical Care Survivors in a Ward Setting

**DOI:** 10.1111/jocn.70460

**Published:** 2026-07-24

**Authors:** Sarah Vollam, Melanie Gager, Hilary Thomas, Peter Watkinson, Natalie Pattison

**Affiliations:** ^1^ Nuffield Department of Clinical Neurosciences University of Oxford Oxford UK; ^2^ NIHR Oxford Biomedical Research Centre Oxford UK; ^3^ Royal Berkshire NHS Foundation Trust Reading UK; ^4^ Centre for Research in Public Health and Community Care, School of Health, Medicine and Life Sciences University of Hertfordshire Hatfield UK; ^5^ Adult Intensive Care Unit Oxford University Hospitals NHS Foundation Trust Oxford UK; ^6^ School of Health, Medicine and Life Sciences University of Hertfordshire Hatfield UK; ^7^ East and North Herts NHS Trust Stevenage UK

**Keywords:** critical care, ICU recovery, otherness, rehabilitation, ward care

## Abstract

**Aims:**

Discharge from an intensive care unit (ICU) to the ward has long been recognised as a difficult and dangerous time for patients. This study aims to explore the experiences of patients, family members and staff of ongoing care following discharge from ICU to the ward.

**Design:**

An exploratory qualitative interview study, part of a mixed methods research project exploring post‐ICU ward care to inform practice changes to improve outcomes for critical care survivors.

**Methods:**

Semi‐structured interviews were conducted with 55 purposively sampled patients, family members and multi‐professional staff between 2017 and 2018 at three NHS trusts in the UK. Data were analysed thematically.

**Findings:**

Three main themes were identified: *Being wardable* discusses the tension between perceptions of readiness for ICU between ICU and the ward staff; *Post‐ICU patients as other* analyses the characterisation of otherness of post‐ICU patients in comparison with other ward patients; and *Fear and anxiety* considers the impact of this perceived otherness on both patients and staff.

**Conclusion:**

The term *wardable* is used by staff to indicate the suitability or otherwise of patient transfer. There is a tension between being deemed wardable from an ICU and ward perspective. This exacerbates the perception of otherness and compounds the fear and anxiety related to post‐ICU care experienced by both patients and ward staff.

**Implications for Patient Care:**

By recognising the tension between being ready for ICU discharge (not requiring organ support) and being wardable (having care needs which can be fulfilled on the ward they are being discharged to), clinicians may better support patients during this transition of care. This has the potential to improve outcomes for post‐ICU patients, as well as improve the experience by both patients and the staff and family members caring for them.

**Reporting Method:**

The COREQ reporting checklist was used in the reporting of this manuscript.

**Patient and Public Contribution:**

Patients and public were involved throughout the REFLECT project, from design through to dissemination. This included advising on the approach to patient participants and supporting dissemination of results via social media.

**Trial Registration:**

ISRCTN: 14658054

## Impact

- What problem did the study address?
  ○ Patient distress following transfer from ICU to the ward has long been recognised but the reasons underlying this have not been explored.
- What were the main findings?
  ○ This study found a dissonance between ICU and ward perceptions of patient readiness for the ward—termed *being wardable*. Post‐ICU patients were perceived to be *other* than general ward patients due to higher care needs and workload, which staff struggled to deliver. This otherness resulted in fear and anxiety for patients, their families and the staff caring for them.
- Where and on whom will the research have an impact?
  ○ This research has the potential to inform changes to the support provided to patients transferred from ICU to the ward, improving outcomes for patients, as well as the experience of patients, their families, and ward staff.
- What does this paper contribute to the wider global clinical community?
  ○ An understanding of the experiences of patients discharged from ICU, their families and the staff who care for them.
  ○ An exploration of the underlying causes of relocation stress following transfer from ICU to the ward.
  ○ An exploration of the term wardable, and how perceptions of this readiness for transfer from ICU to the ward differ between settings.

## 1 Introduction

The proportion of patients surviving critical illness has been increasing steadily for many years, due to improvements in management (Paul et al. 2022). In recent years this has shifted the focus for research and innovation in critical care beyond survival to intensive care unit (ICU, also referred to as ITU—intensive therapy unit) discharge, to consider recovery and rehabilitation following critical illness, termed the third revolution in critical care (Iwashyna and Speelmon 2016). Discharge from an ICU to the ward is a significant step in recovery from critical illness and an important transition for patients. However, it has long been recognised that the physical and psychological effects of critical illness can be profound and commonly persist long after ICU discharge (Yuan et al. 2021). Moreover, the societal impact of critical illness recovery is wide‐ranging, from inability to return to former roles and functions, such as involving work, to increased healthcare burdens and wider social determinants of health (Howard et al. 2024).

## 2 Background

The negative psychological impact of the move from ICU to the ward was first recognised over fifty years ago (Dominian and Dobson 1969). Subsequent studies have explored the patient and, to a limited extent, family perspectives of the immediate period following transfer from ICU to the ward (Bench and Day 2010; Cullinane and Plowright 2013; Forsberg et al. 2011). The impact of moving from a highly technical area with 1:1 nursing and close monitoring to a ward where nurses are far less visible and accessible has been termed transfer anxiety or relocation stress (Yumei et al. 2024). Few studies have looked beyond this psychological impact to explore factors which may contribute to relocation stress or to consider the patient experience past the immediate transfer period. Where this has been considered, exploration is very limited but has identified factors including physical symptoms such as reduced mobility, pain, weakness, loss of appetite and poor sleep (Strahan and Brown 2005), leading to feelings of vulnerability (Herling et al. 2020). In contrast, Field et al. (2008) developed consideration of underlying factors for relocation stress further, challenging the attribution of transfer stress to the psychological impact of a change in environment. They identified several causes of distress, such as poor basic care provision, lack of understanding of the limitations of post‐ICU patients, and the inappropriateness of the ward environment for post‐ICU patients. They concluded that, although relocation stress has been a useful way of conceptualising the experience of transfer from ICU to the ward, there is a need to understand the underlying causes of this distress and educate staff to understand and address patients' experiences.

Several studies have examined the perception and concerns of ward nursing staff receiving patients from ICU (Enger and Andershed 2018; Kauppi et al. 2018; Cognet and Coyer 2014; Häggström et al. 2018). These included perceptions that post‐ICU patients require a high workload and/or care outside of their scope of competence (Enger and Andershed 2018; Kauppi et al. 2018). Little further exploration of these concepts was undertaken within these studies, and no study examined the perspectives of staff other than nurses. Thus, whilst relocation stress has long been recognised, and there is some awareness of the underlying factors which may contribute to this from both patient and staff perspectives, this only presents the patient and nurse perspectives of the early period after transfer from ICU to the ward.

This current study draws on the concept of otherness in considering the process of transfer from ICU to the ward. Otherness was first conceptualised by the French philosopher Levinas describing the *other* as giving rise to spontaneous moral responsibility (Levinas 1974). This responsibility is described as particularly pertinent when the *other* is vulnerable (Peperzak 1993), which is commonly the case for patients discharged from ICU. Vulnerability centres not only on physical attributes of the individual, or psychological sequelae, but also on the environment to which the ICU patient is being transferred. Omissions in ward nursing care, well documented across Europe, contribute to a new layer of vulnerability (Herling et al. 2020).

## 3 The Study

### 3.1 Aim

The aim of this paper is to explore the experiences of patients, family members and staff of ongoing care following discharge from ICU to the ward.

## 4 Methods

### 4.1 Design

This exploratory qualitative study formed part of a wider mixed methods research project—the REFLECT (REcovery Following criticaL carE Treatment) study (NIHR PB‐PG‐0215‐36,149), which aimed to explore post‐ICU ward care to inform change in practice to improve outcome for critical care survivors. The project used three methods to collect data: Retrospective Case Record Review; in‐depth case analysis; and semi‐structured interviews. The protocol was prospectively registered (ISCTRN 14658054) and published (Vollam et al. 2019), and results of the other approaches have been reported (Gustafson et al. 2021; Vollam et al. 2022, 2021). This paper draws on data from the qualitative interviews within the REFLECT project. The COREQ reporting checklist was used in the preparation of this manuscript (Tong et al. 2007).

### 4.2 Study Setting and Sampling

Data were collected at three NHS sites in England between 2017 and 2018, prior to the COVID‐19 pandemic. Sites were selected to provide variation in size of hospital, ICU, and post‐ICU support infrastructure (see Table 1 for characteristics of each site). All sites were situated in south/central England to facilitate site access. A full account of methods can be found in the prospectively published protocol paper (Vollam et al. 2019).

**TABLE 1:** Site characteristics.

Site	A	B	C
Hospital beds	1049	650	538
ICU beds	22	14	6
HDU provision	Integrated level 2 beds within ICU	Yes, separate to ICU	Yes, separate to ICU
Clinical services	Dedicated ICU physiotherapy team Dedicated ICU dietician On‐site microbiology service On‐site TPN service Nurse‐led ICU follow‐up service 8 am‐6 pm: All patients with > 4 day ICU stay visited until deemed well enough to be discharged from the service (often within one to two days)	Dedicated ICU physiotherapy team Dietetic service covering ICU and wards On‐site microbiology service Off‐site TPN service Nurse‐led ICU outreach service 24/7: All patients with > 4 day ICU stay, daily visit until deemed well enough to be discharged from the service (often within one to two days). Plus weekly visit from the Rehabilitation after Critical Illness team	Physiotherapy team covering ICU and wards Dietetic service covering ICU and wards Off‐site microbiology service Off‐site TPN service Nurse‐led ICU outreach service 24/7: All patients > 48 h ICU stay, daily visit until deemed well enough to be discharged from the service (often within one to two days)
Ward layouts	Site 1: large distance between ICU and wards. Most wards a mixture four‐bedded bays and few side rooms Site 2: acute wards in close proximity to ICU and wards with some four‐bedded bays but predominantly side rooms, some side rooms only	One large site Large distance between ICU and most wards. Most wards a mixture of four to six‐bedded bays and side rooms	One site Acute wards in close proximity to ICU. Most wards a mixture of four‐bedded bays and side rooms

### 4.3 Sample Size

The anticipated sample size required for this study was based on the concept of information power (Malterud et al. 2016), aiming to access a wide range of professions and experiences (e.g., clinical speciality and duration in role for staff, and clinical speciality, length of stay on ICU and care needs after discharge for patients) across three sites. Considering the fairly broad topic encompassing the whole post‐ICU ward period, an approximate sample of 30 staff and 20 patients and family members was anticipated to be required. Towards the end of data collection, adequacy of the anticipated sample was considered based on data saturation. Recruitment ceased when the research team agreed data saturation was achieved, that is, no new codes were being generated in analysis.

### 4.4 Inclusion and Exclusion Criteria

Staff were eligible to participate if they worked with patients transferred from ICU to the ward at the participating site. There were no exclusion criteria. Staff recruitment was based on purposive maximum variation sampling both between and within groups, aiming to access a broad range of professions within the multi‐disciplinary team and levels of clinical experience. Staff were recruited by research nurses at each site using several approaches, including advertising locally through trust communication boards and newsletters, clinical managers sending e‐mails to staff, and snowballing sampling targeting specific professional groups not yet represented in the sample.

Patients were eligible to participate if they had been transferred from ICU to the ward and were asked if they had a family member they would like to participate in the study. Participants were excluded if they did not speak English. Patient and family member recruitment was informed by guidance from a local group of patients and their families with experience of critical illness. This included a discussion of the timing of approach and interviews. Patient representatives advised that conducting interviews too soon after hospital discharge would be too difficult for patients and family members, as this was a period of significant adjustment. This needed to be balanced with the risk of reduced recall if interviews were conducted too long after the event. Routinely, patients discharged from an ICU are invited to a follow‐up clinic at 3–6 months following discharge from ICU to review ongoing rehabilitation and identify any lasting physical or psychological effects of critical illness, in line with national guidance (FICM 2022). This was agreed with the patient group to be the optimal timepoint for interviews. Patients were approached via follow‐up clinics at each site, with information about the study being sent out with their appointment letters. Postal consent was offered for those who did not wish to attend the follow‐up clinic. Purposive sampling was also employed for patient participants, aiming to include a range of ages, backgrounds and experiences of recovery from critical illness. Interviews were offered individually or with a family member present (as a dyadic interview). Recruitment of bereaved family members was also planned to offer insight into the care of those who did not survive their critical illness. The planned approach for recruiting bereaved family members included screening patient lists and sending invitation letters to next of kin 6 months following bereavement or recruiting via ICU support groups.

### 4.5 Data Collection

Two female researchers conducted the semi‐structured interviews (SV and MG), using a topic guide with questions informed by current literature and expert opinion. Both researchers were experienced in conducting qualitative interviews. SV has a critical care nursing background but was not working clinically. She was known to some clinical staff at one site but did not work with them. MG has a critical care nursing background and was working clinically at one site, so was known to some clinical staff. Interviews were held either by telephone or face‐to‐face in a quiet room away from the clinical area (for staff) or near the follow‐up clinic (for patients). No‐one other than the participants and researcher was present during interviews. Interviews were audio recorded, transcribed verbatim, anonymised and assigned a study number. Transcripts were not returned to participants for checking.

### 4.6 Data Analysis

Analysis was undertaken on paper and using NVIVO software (lumivero.com) following the seven steps of reflexive thematic analysis, as described by Braun and Clark (Braun and Clarke 2019). In the first step, familiarisation, SV read and re‐read the transcripts, then commenced initial coding (step two). In the third step (searching for themes), SV discussed these initial codes and how they were potentially linked into broader early themes with NP and HT. This included reviewing code books and drawing mind maps. In step four (reviewing themes) these themes were developed through constant discussion and consensus of the research team, until the final themes were defined and named (step five). Anonymised quotes are presented below to Illustrate the themes, along with site and participant descriptor(s) (e.g., staff group, patient or family member) (step 6).

### 4.7 Ethical Considerations

Ethics approval was granted by Wales Research Ethics Committee 4 (reference 17/WA/1039). All participants received written information about the study and were assured that participation was voluntary and anonymous. Written informed consent was obtained for each participant. A standard operating procedure was developed to guide the researchers in how to deal with any disclosure of poor care, together with a distress protocol. This included signposting to support groups, the local follow‐up clinic and external patient support charities for patients, and escalating any concerns regarding poor care or professional accountability to the local NHS trust for staff. No issues were identified during data collection.

### 4.8 Rigour and Reflexivity

The clinical background of both interviewers (SV and MG) requires careful consideration in terms of reflexivity. Both had critical care nursing backgrounds, and one worked as a Rehabilitation After Critical Illness specialist nurse. One member of the research team involved in analysis (NP) was also a Clinical Lead for Critical Care Recovery Services. These backgrounds had the potential to shape both the questions asked in interviews and the way in which participants framed their answers. In addition, their experiences may have shaped subsequent analysis and interpretation. The team discussed the potential impact of the researchers' backgrounds and how to manage this to mitigate against bias. Several steps were taken to ensure trustworthiness and credibility. As one researcher worked clinically at one site and was known to some participants, the other researcher conducted some of the interviews at this site to offer an outsider perspective. In addition to a list of open questions and prompts, the topic guide included sections for reflexive notes, prompting the researcher to reflect on biases, assumptions and internal or external factors which may have influenced the interview. These were utilised during analysis to provide context to the interviews. Mind maps were used to develop and record synthesis during analysis. Research diaries were kept by the lead researcher. Throughout analysis, regular group discussions were held, exploring initial and developing codes and themes, and finalising themes.

## 5 Findings

### 5.1 Characteristics of Participants

Interviews were held with 30 multi‐professional staff members, representing a wide range of professions, including ward nurses (*n* = 13), three physiotherapists, three specialist registrars (mid‐grade doctors), four foundation year doctors (newly qualified doctors), a dietician, five critical care outreach/follow‐up team nurses and one clinical support worker. No medical consultants (senior doctors) were available for interview. A total of 26 patients (*n* = 18) and family members (*n* = 8) were interviewed. Interviews were held with 10 individual patients, seven patient/family member dyads (with three wives, two husbands and two daughters) and one bereaved family member (wife of the patient). The duration of interviews varied between 12 and 65 min (mean 35 min).

### 5.2 Theme Overview

Findings are presented under three interconnected main themes. *Being wardable* discusses the tension between perceptions of ICU and the ward on what it means to be wardable—that is, ready for transfer to the ward. *Post‐ICU patients as other* analyses the ways in which those discharged from ICU to the ward are perceived as different to other ward patients, the implications of these perceived differences for clinical care and how this contrasts with the concept of being wardable. *Fear and anxiety*, the final theme, considers the impact of this perceived otherness and readiness for the ward on both patients and staff.

### 5.3 Being Wardable

Readiness for transfer to the ward was an important issue for participants. Staff members from different professional backgrounds, including doctors, ward nurses, physiotherapists, and critical care outreach nurses, characterised the readiness to be moved from ICU to the ward as *being wardable*.

Interviewer And expecting those patients from intensive care, what are those patients like?

Respondent It can vary quite a lot. I mean you expect some patients that come from intensive care to be still quite unwell but stable enough to be wardable. Clinical support worker, staff interview 3, site B

Although commonly used by staff, patient and family members did not use the term wardable, but did describe a significant change in care provision between ICU and the ward which they did not feel they fit into. The transfer from ICU to the ward was characterised by one patient as ‘being let loose in the wild again’, suggesting a move away from the role of critically ill patient, for which they may not have felt ready.

Obviously I was not ready to be discharged or the medication I was given at the point of discharge was not correct for me to be let loose into the wild again. Patient 3, site B

Both staff and patients identified tension between being deemed ready to leave ICU (i.e., no longer needing organ support, which commonly defines ICU care) but still requiring a level of care that the ward system was unable to meet.

[O]ne of the big frustrations is that patients needed intensive nursing […] but there being nowhere to get that but ITU, and then ITU not being able to offer that because they needed it for people who need level 3 organ support. Foundation year doctor, staff interview 6, site A

This tension had profound consequences both for patients, who described the impact of their needs not being met on the ward, and staff, who were aware of their inability to deliver the standard of care they would like to their patients. The underlying reasons for this will be explored in detail below.

[B]ut they also did keep me in a wet bed and also because of the antibiotics they were going through me and I had very bad diarrhoea and there was one time they actually left me in the bed with the diarrhoea and I was literally covered … and I ended up getting really bad sores, pressure sores. Patient 3, site A

He was left uncomfortable for a long time […], I think, too long. I don't know but I think there weren't enough staff on and it was a terrible time. Family member 7, site B

There was a consensus among staff that patients discharged from ICU may still be unwell, but needed to be relatively physiologically stable to be wardable that is, their vital signs were within, and expected to remain within, normal ranges. Clinical instability requiring continuous monitoring was highlighted as an aspect of care more appropriately provided on ICU than the ward.

We've got a cardiac monitor on the ward that we used when we need to if we're concerned. We don't like to keep people on them continuously because I always feel if they're requiring that all the time then they shouldn't really be on the ward is my feel for it. Ward nurse, staff interview 7, site B

Staff recognised that even if a patient appeared wardable at a particular point in time, they were still at high risk of deterioration, especially if organ support had been recently stopped. ICU staff described trying to keep patients in ICU for longer if they were perceived at high risk of deterioration, taking into consideration fluctuating levels of ward staffing (i.e., at the weekend).

[Y]eah so I'm involved in you know, regularly involved in decisions about discharge so I'll say if a patient's ready to go and that's kind of a multifactorial decision so the first question is I'll ask myself is are they physiologically stable? As in not only are they stable now but are they likely to stay stable for the next 24/48 hrs? And I'll do a basic risk assessment on that and if they are high risk to re‐deteriorate then I won't discharge them. So I want physiological stability plus the expected projection of stability. ICU registrar, staff interview 10, site A

We're quite keen if a patient is wardable but we have room to keep them in ICU, maybe over a weekend, we would perhaps keep them in intensive care because we know that over the weekend it's skeleton staffing and obviously intensive care patients always have the potential after critical illness to deteriorate. Critical Care Outreach Nurse, staff interview 7, site B

Ward staff described the consequences of not considering potential stability over several hours, for example transferring a patient soon after medication to support blood pressure was stopped, before ongoing stability was ascertained.

I think sometimes [ICU staff] withdraw the help that they need with the blood pressure and stop all these drugs and [the patient] can hold their own for a little while and they think they're wardable now and then they'll [do a ward] round and a couple of hours later all their obs[ervations] are to pot again so that's one of the things we've faced on the ward sometimes. Ward nurse, staff interview 7, site C

Staff commonly described high bed occupancy as the reason for patients who were not wardable being discharged from ICU, with decisions being made based on balancing the needs of multiple patients. It was also recognised that this balance was not always achievable as the following staff comments illustrate.

I think sometimes ICU have got their own bed status and we've got our own bed situation and stuff and obviously I'm talking about it from our point of view I've never actually worked in ICU but I know that obviously they could suddenly have some really sick patients down in A&E that need to be tubed or that need an urgent admission and they might have a fairly stable respiratory patient that's on some IV [intravenous] antibiotics that was de‐cannulated the day before that perhaps from a consultant point of view could be wardable. Ward nurse, staff interview 7, site C

Interviewer What one thing worries you most about the transfer of patients from ITU?

Respondent That they're ready to come.

Interviewer What does that mean?

Respondent Medically well enough to be coming down to the ward. I think especially when at times there has been high pressure not only on our beds but on ICU beds that patients are tending to be moved, I almost hesitate to say it, but on a wing and a prayer sometimes. Physiotherapist, staff interview 2, site B

Ward staff suggested that ICU staff may not always be honest or transparent about this. Given the identified mismatch between perceptions of being wardable from ICU and ward perspectives this may indicate a lack of awareness of the ward environment from an ICU perspective rather than dishonesty.

I suppose I always worry about whether I can look after them and are they wardable or are they pushing them out because they need an ITU bed and can I look after them … if you probably ask an ITU nurse some of the patients that get discharged aren't completely wardable yet but they're the most wardable so they're the ones that go. Ward nurse, staff interview 10, site C

We have patients that come from intensive care units from other hospitals and sometimes they're not quite as honest as they should be about the patient's condition and we have had a few issues in the past where we've been told the patient has been stepped down, they're wardable but when the patient gets here they're actually a bit more unstable and possibly even shouldn't have been transported. Clinical support worker, staff interview 3, site B

It was notable that the only staff member to express an opposite opinion—that patients were always appropriately stepped down from ICU—was one foundation year doctor who may have had limited exposure to managing post‐ICU patients, better support mechanisms within their local team, or geographical proximity to ICU which may have resulted in close collaboration or communication between the settings.

From a patient perspective, the move to the ward suggested expectations that did not always match with their physical condition. Despite these concerns, some patients described assuming that the decision to move them from ICU to the ward must be the right one for their care, placing trust in healthcare professionals supporting them.

I think it's also that but also the fact when you went from ICU to the ward you are made to feel like you are normal so that when you leave hospital… There were times I would say Dad, you have to stop you've spent a month in ICU and you can't expect yourself to be able to do these things. I think because he went onto the ward and you're made to feel normal you kind of get into that whole okay I guess I can carry on afterwards. Family member 2, site B

[Y]ou've got to manage. Someone has made the decision that I'm strong enough to be in [ward name] on my own and not twenty four hour care therefore the nurses I'm sure thought he's okay to be there and I probably was although it was a frightening experience. Patient 6, site A

Staff commonly used the term *wardable* to convey the commonality and impact of patients not being wardable when they arrived on the ward, either due to occupancy pressures or lack of understanding of the ward environment. Whilst patients and family members did not use the term *wardable*, they also described a lack of readiness for transfer. Patients and staff described *being wardable* as being, and predicted to remain, physiologically stable, not too reliant on help from nursing staff and not requiring specialist skills to manage their care needs.

### 5.4 Post‐ICU Patients as Other

Both patients and staff described being aware of how different the care needs of those transferred from ICU were compared with the general cohort of patients on the ward, resulting in them being perceived as other in this context.

From the patient perspective, this was commonly framed as physical dependence that is, requiring help to move. Patients and family members described feeling that ward staff underestimated how weak they were following critical illness, consequently expecting them to be able to do more for themselves than they were physically able to do.

I found it difficult in some respects that some people were, I think, physically fitter than what I was at that time … Patient 1, site A

I was so weak but I was told I'd be alright and I fell and hurt myself on my knee and whatever. They were not very sympathetic in so much as they just underestimated my weakness and thought I could just get up by myself which of course I couldn't. Patient 2, site C

Patients suggested this may reflect a lack of awareness from ward staff of the global physical effects of critical illness, and/or a failure of communication, with one patient questioning whether this was handed over between clinical settings.

The other issue that we had was the physio[therapist]s, wasn't it? Because they were giving him things to do but they didn't seem to realise how weak he was, they were just giving him exercises for his knee rather than taking into account what else he'd been through and wasn't strong enough or able to do what they were suggesting. Family member 4, site B

[A]s soon as you go from ICU into the ward you get lost in the mix again so it might not be something you do but it wasn't observable to me, but there is something there that says this patient is a little bit different because they've just come out of ICU and therefore might need a little bit more or expecting a little bit more care and attention than everyone else. Patient 3, site B

From a staff perspective, several characteristics were identified which contributed to the otherness of post‐ICU patients. In contrast to patients' focus on physical dependency, these characteristics included being more acutely unwell, at higher risk of deterioration and having complex care needs such as tracheostomy management and nutritional support, as well as being physically dependant and psychologically traumatised. In this context, staff discussed their anxiety about how post‐ICU patients would manage on the ward.

Sometimes I think oh my god, how are you going to get by on the ward … because they're so weak and fragile and frail … Critical Care Outreach Team/follow‐up nurse, staff interview 4, site B.

And so patients who are physiologically well leaving ICU, having recovered from organ failure, still have complex care requirements be they nutritional, fluid related, medication related, and not least psychological. And so you have a patient who's recovered from their [physiological] insult and but still has complex issues. ICU Specialist Registrar, staff interview 10, site A

I'm definitely always more worried about a patient who's come from intensive care than a regular general medical patient just because they've physiologically been through a huge amount and may not have the same reserve and I do think they get sicker quicker. Specialist Registrar, staff interview 5, site B

There was also consensus among staff that not all post‐ICU patients had characteristics of otherness, and that some more complex ward patients may be at similar risk of deterioration than those discharged from ICU. Participants therefore suggested patients should be considered on an individual basis.

Um, so our ITU patients do frequently deteriorate, but, I think, they are just very complex, and so, do they deteriorate any more than our very complex trauma patients? Potentially not a lot. Ward nurse, staff interview 1, site A

I'm never worried receiving a patient from ITU, I only worry when they start deteriorating, no I'm never worried. I just focus on the patient. Ward nurse, staff interview 6, site C

Staff described ways characteristics of otherness were managed within the constraints of the ward environment. This included placing post‐ICU patients in observation bays (beds closest to the nurses station) so they were more visible to staff.

ITU [patients] always come into that acute bay, they never go into the off bays … we still prefer to keep them there just for that closer monitoring and probably as well for their reassurance to be honest but mainly for our reassurance so that we know they're going to be seen that little bit extra. Ward nurse, staff interview 5, site C

Reflecting the perception that post‐ICU patients are generally at higher risk of deterioration than other patient groups on the ward, nurses commonly described taking vital signs measurements more frequently for post‐ICU patients than other ward patients, both to establish a baseline to measure against, and ensure prompt identification and action if a deterioration were to occur.

With step‐downs [patients transferred from ICU to the ward] obviously, they [vital signs measurements] need to be done a little more often to make sure that they're still stable and maintaining what their normal baseline would possibly be. Clinical support worker, staff interview 3, site B

Staff identified that high care needs may require nurses with specific skills and experience which they and their colleagues may not have, and therefore receiving patients from ICU may have implications for staffing and skill mix on the wards.

[A]nd then you do get some that come down that are very complex that have got all sorts of needs and that's where it's important that you look at your staffing for the shift and you make sure that you've got staff that can deal with, for example they might have a PEG [permanent feeding tube] in, they might have a trache[ostomy], they might be on NIV [non‐invasive ventilation] and they might be on lots of different things. Ward nurse, staff interview 7, site B

[I]f they've got drips and drains and PCAs [patient‐controlled analgesia] and things it's got to be somebody who's been qualified for a while and has got those skills … Ward sister/charge nurse, staff interview 5, site A

Not all staff participants agreed that post‐ICU patients required specialist skills. Anxiety about the need for extended skills might reflect the awareness of staff that they struggled to offer the high quality care they would like due to their high workload on the wards.

It was – I don't know … understaffing, but you need people with skills just to do the ordinary nursing, it isn't rocket science and it was that [understaffing] that I thought didn't help. Family member 7, site B

I think it's mostly time pressure … and some skills and knowledge. I think that if you put an ITU nurse in that situation then they would probably have a better idea of what the most important things to prioritise were. Umm … but they still won't probably be able to do it all because of time pressure. Foundation Year doctor, staff interview 6, site A

Post‐ICU patients were identified as other than general ward patients by staff, patients and family members. Patients viewed otherness in relation to physical dependence, and the consequences of this dependence on the ability of staff to meet their care needs. Staff perceived this as broader, encompassing characteristics such as higher acuity of illness, risk of deterioration and advanced care needs not usually required by other patients on the ward. Staff discussed several strategies to manage these characteristics of otherness within the ward setting but both staff and patients identified that accommodating these greater care needs within the ward environment was challenging, with clear examples of patients' needs not being met.

### 5.5 Fear and Anxiety

Despite the measures taken by staff to accommodate the characteristics of otherness, it was clear from interviews with both ward staff and patients that this was not sufficient to prevent the impact of otherness on patient care. Patients identified profound consequences of the limitations of ward‐based care, describing their basic care needs as not being met, leading to a sense of vulnerability. Vulnerability was previously identified in studies of relocation stress (Bench and Day 2010), although in this study patients directly linked this to the consequences of being a physically dependent patient in an area unable to meet their needs, indicating a physical rather than psychological response to the change in location, as identified by Field et al. (2008).

I know when I moved to [ward name] there was a feeling of fear that I'd had twenty four hour care, there was someone beside me in an open ward and suddenly my only point of safety was a button that I didn't really have the strength to press that could easily have fallen out of my hand and yes it was quite terrifying. Patient 6, site A

If the patient had got lots of needs and they're going to a busy ward where the ward won't be able to meet those needs because that is not seen as a reason for the patient to stay in ICU because I know that the ICU bed is a scarce resource, but you see this very vulnerable patient who has got lots of needs and they're going to an area that maybe is not going to meet those needs. CCOT/follow‐up nurse, staff interview 4, site B

Staff were acutely aware that the characteristics of otherness of post‐ICU patients challenged their ability to provide the level of care they would like. This manifested as concerns about their clinical skills, workload and the broader system of care. Staff described feeling clinically vulnerable due to these limitations. In particular, newly qualified doctors described anxiety about managing patients recently transferred from ICU. Anxiety was described as relating to their perceived high risk of deterioration, which they feared they would not be able to manage safely on their own, and the lack of detailed handover about the patient.

They might be recovering from severe infections and they're at a limbo state where they might potentially get worse or they could be getting better and so you need to be able to identify which way they're swinging. They're just not as stable as a general inpatient on the ward. Foundation Year doctor, staff interview 13, site C

The patients who really gave me the heebie jeebies were people with medical co‐morbidities who'd gone to ITU for medical care … Foundation Year doctor, staff interview 6, site A

In contrast, continuing their discussion of appropriate discharges from ICU, one foundation year doctor suggested they did not feel worried about receiving patients from ICU, as they were well supported and able to contact ICU if the needed help.

I never felt that we in an unsafe position having the patient back on to the ward. Foundation Year doctor, staff interview 14, site C

Ward staff commonly acknowledged the potential for post‐ICU patients to be anxious on the ward, due to their perceived otherness. To address this, some staff members described being direct with patients about the reality of care provision on the ward, recognising that they may not be able to meet the patients' expectations had of the care they would receive. Other staff described framing this in a more positive way, as a sign that the patient was recovering.

I think we just have to be quite brutally honest with them really and say we are obviously going to monitor you still closely however I can't stand at the end of your bed when I've got up to another fifteen patients … Ward nurse, staff interview 5, site C

[J]ust explain to them, make them aware that they no longer need that level of high care and that they are getting better. It will make the patient feel a bit more positive. Well, I was having 1:1 nursing care and now I'm not so I must be getting better. Ward nurse, staff interview 9, site B

However, in the absence of clear communication about how care would be managed, and a safety net to reassure patients they could get the help and information they needed, this risked causing patients (and their family members) further distress and anxiety.

I know when I moved to [ward name] there was a feeling of fear that I'd had twenty four hour care, there was someone beside me in an open ward and suddenly my only point of safety was a button that I didn't really have the strength to press that could easily have fallen out of my hand and yes it was quite terrifying. Patient 6, site A

Patient Yes I was okay. I think it was worse for my wife than it was for me.

Interviewer Why do you say that?

Patient Well because she wasn't getting any information and she needed to feel comfortable but just so that she knew what was happening to stop her worrying. Patient 2 & Family member 1, site B

This theme explored the consequences of post‐ICU otherness for both patients and staff. Patients recognised that their care needs were not being fully met on the ward due to their otherness, and this made them feel anxious and vulnerable. Staff also recognised this limitation in their ability to meet these high care needs, resulting in anxiety regarding their clinical skills and discomfort at not being able to provide the level of care they wished.

## 6 Discussion

The three themes presented in this paper explore the concept of being *wardable*, what this means for patients and staff, and how this articulates with the concept of otherness. Fear and anxiety run through these two themes as a consequence of otherness and highlighting the question of whether patients are really *wardable*. This is reflected in the anxiety regarding the readiness of patients to be transferred to the ward and worsened by the pressures of bed occupancy, skill mix and staffing. These two concepts will be discussed below, along with the consequent fear and anxiety they bring for all involved in post‐ICU care.

### 6.1 Being Wardable

In considering the complex process of transition from ICU to the ward and the establishment of care in the latter, we argue for a new concept of *being wardable*, drawing on a phrase used by clinical staff in the interviews, noticeably deployed more in the breach than the observance, that a patient was indeed in a fit state for transfer to the ward. We argue that *being wardable* reflects both professional assessment of the patient and their needs, and its counterpart in patients' and family members' experiences and perceptions.

*Being wardable* as professional assessment may be concerned with: the patient's condition; ward staff abilities (including structurally influenced staffing availability and individual skills‐based abilities) to meet the patient's needs including structurally influenced and individual skills‐based abilities; and the temporal context of work flows, such as at weekends when a patient may be retained in ICU due to a combination of lower ward staffing levels and the ever‐present risk of deterioration in the patient's condition. While not a phrase used by patients and family members, the constituent parts of *being wardable* have equivalents in: concern about the timing of admission to the ward given the patient's condition; perceptions of staff abilities to meet the patient's needs; and a more general understanding that the ward would provide less support together with concern about whether support was sufficient.

While patients and family members did not often question the decision for transfer from ICU to the ward (‘they know what they're doing’), concern was expressed about whether support was adequate for patients in their condition. *Being wardable* therefore, combines judgement and process in transfer of patients from a situation that provides close clinical support for patients similarly placed, to a situation in which their clinical condition and risk‐laden prognosis set them apart from ward peers and renders them ‘other’. Both staff and patients and their families identified this otherness and expressed concern amounting to fear and anxiety as a consequence.

### 6.2 The Implications of Otherness

In this study, both patient and staff participants clearly identified a perception of post‐ICU patients as other than general ward patients, due to their care needs exceeding what the ward was able to provide. This made delivering adequate care challenging, potentially impacting recovery as well as contributing to the fear and anxiety for both patients and the staff caring for them described in the third theme. This develops the findings of previous qualitative studies, where elements of otherness were identified. Previous studies have identified both patients and nurses struggling with the transition from ICU to the ward, termed relocation stress from the patient perspective and a component of the fear identified in the third theme (Yumei et al. 2024; Strahan and Brown 2005). This study builds on the work with patients by Field et al. (2008) who argued that relocation stress should not be seen as an inevitable consequence of transfer from ICU, but that elements of care may contribute and should be further researched. In this study, multi‐professional staff and patient and family members described multiple characteristics which contributed to this characterisation of otherness of post‐ICU patients. Staff described this in stark terms, identifying a variety of attributes such as acuity, high dependency, complexity of care needs and associated high workload. Advanced clinical skills, experience and time were all aspects identified as essential in managing *otherness* across all staff groups but particularly starkly for the most junior doctors. In common with the findings of this study, previous studies found nurses viewed post‐ICU patients as increasing their workload and requiring advanced skills they may not possess, resulting in anxiety at receiving a patient from ICU (Enger and Andershed 2018; Kauppi et al. 2018). To our knowledge, no similar studies have explored the perspectives of staff groups other than nurses.

Jebran (2014) and Nortvedt (2003), both described the conflict between meeting the needs of the individual patient and limitations of resources to provide care, clearly identified in this current study. In the context of post‐ICU care, the vulnerable *other* exists alongside further ward patient others, who also have care needs. This is reflected in the anxiety experienced by staff in trying to balance meeting the high care needs of post‐ICU patients alongside the usual workload of patients on the ward. Whilst Jebran (2014) framed this argument as one of futility versus rationing, in this study of post‐ICU patients, rationing workload was not related to futility, but to the rarity of the high needs of post‐ICU patients. (Clifton‐Soderstrom 2003) identified that where there is conflict between meeting individualised needs and the needs of the third that is, the wider cohort, this may result in significant moral distress for staff. This was clearly identified in this study. Where staff were confronted with the needs of vulnerable post‐ICU patients, they described struggling to meet these needs whilst also ensuring safety and care of the wider ward cohort, resulting in distress and anxiety.

In contrast to previous conceptualisation of *othering* as discriminatory due to differences (Jebran 2014), Canales' advocates for inclusionary othering, recognising what it is to be *othered* (Canales 2000). This emphasises the importance of identifying the *otherness* of post‐ICU patients, as doing so allows staff to understand the challenges of managing post‐ICU patients within their workload and plan strategies to manage this. (Ryan 2012) also suggested that where the *othered* person is recognised and helped they may be able to adapt to their *otherness*, but will remain isolated and vulnerable if not. By identifying where the challenges for these *other* patients lie, changes may be made to the system to support care provision and relieve the fear and anxiety experienced by all those involved.

### 6.3 The Wardable Continuum

Mackintosh and Sandall (2016) have described the consequences of the deteriorating patient in the context of Strauss et al.'s trajectory of illness, highlighting that errors in managing deterioration are often due to organisational issues rather than human error (Strauss 1997). Strauss et al. described the way hospital wards are established to manage the routine needs of their usual patient cohort, a process they termed the shape of the ward (Strauss 1997). The shape is characterised by several factors, such as the number and expertise of the staff, and easy availability of specific drugs and equipment commonly needed. Admitting patients who do not fit the shape of the ward challenges the routine trajectory of care and risks errors and failures in care delivery, as well as significant fear and anxiety for patients and staff. Strauss (1997) recognised that when trajectories of illness become unpredictable, mistakes and omissions are more likely, resulting in risk to the patient. Where the patient's needs do not fit within the shape of the ward, this is likely to be exacerbated. In our work we found this to be further compounded by the perception that post‐ICU patients may be transferred from ICU when still unstable and/or at high risk of deterioration, suggesting patients may not be in the right place of care at that point in their recovery. This further challenges the ability of ward staff to provide the care needed, resulting in fear and anxiety for everyone involved. It was clear that staff were acutely aware of this, resulting in anxiety about their ability to care for these patients and keep them safe.

Whilst discharge from ICU to the ward is a significant transition, these findings demonstrate that patients discharged from ICU commonly have ongoing high care needs, which may not be manageable within current ward systems of care. This suggests that the transition may not be as distinct as might be supposed. This work demonstrates that there is no clear dichotomy between critically ill and stable patients but rather they are part of a continuum on which they can move backwards as well as forwards. Although discharge from ICU can be a significant step in recovery, where patients are transferred from an environment where their care needs are fully met (due to high staffing levels on ICU in relation to the ward), if they do not fit the shape of the ward to which they are transferred, ward staff may be unable to meet care needs which are other than the general ward population. This may risk a step backwards in recovery. In a prior cohort study, patients discharged from ICU to the ward whilst still physically dependent (i.e., unable to stand and step from bed to chair) were found to commonly take 2 days to return to their discharge level (Haylett et al. 2024). By acknowledging that the characteristics of being wardable can fluctuate, and that no longer requiring organ support is an insufficient indication that a patient's needs can be met on the ward, the ongoing management of critical care recovery may be better supported within current healthcare systems.

The impact of post‐ICU otherness should also be considered alongside the concept of critical care without walls, first described in 2000 (Department of Health 2000). This recognises that there are patients outside of critical care units who are acutely unwell and whose care may benefit from clinicians with critical care expertise. Whilst this is generally framed in terms of patients whose condition is deteriorating that is, those who may be heading towards ICU admission rather than those being discharged from it, it recognises the challenge of managing acutely unwell patients on the ward (Ostermann and Vincent 2023). Although this has been recognised through the introduction of services aiming to support post‐ICU patients—termed transition programmes and usually consisting of nurse‐led teams who visit patients on initial arrival on the ward—these have not been shown to impact patient outcomes such as mortality and length of hospital stay (Österlind et al. 2020). Furthermore, the results of this study suggest there is still work to be done to support those discharged from ICU, and the staff who care for them.

### 6.4 Strengths and Limitations

To aid transferability, this study included sites with varying post‐ICU service provision. Despite these differences in services, the challenges described by patients discharged to the ward, and the staff caring for them, did not differ substantially between sites. This study included perspectives from a wide range of professionals, patients and family members, drawn from three sites with different sized hospitals and post‐ICU support infrastructure. Drawing these perspectives together facilitated contrast between these perspectives but also highlighted the significant commonality in their views. However, data are limited by the recruitment of only one bereaved family member and no medical consultants, due to challenges in recruiting these two groups. In addition, although steps were taken to support participation for patients unwilling or unable to attend their follow‐up clinic appointment, including participating by telephone, it is possible that patients who had not recovered from their critical illness well at the point of invitation may have been less likely to engage with the study. In addition, excluding those unable to speak English posed a barrier to participation and risked losing the voices of marginalised groups.

The interviews were conducted between 2017 and 2018 (prior to the COVID‐19 pandemic). Given the potential for changes in ICU and ward‐based service provision, including ICU discharge thresholds, ward capacity and models of care over time, this may limit transferability to current practice. However, there is no evidence to suggest that post‐ICU care has improved in this time. For example, recently updated Guidelines for Provision of Intensive Care Services in the UK have not significantly changed from earlier versions. Indeed, post‐ICU care is likely to have worsened given the current recognised shortage of staff in the NHS (Royal College of Nursing 2024), as well as an increasing trend in the complexity of hospitalised patients in the UK and internationally (Turner et al. 2026). This is emphasised by the recent National Confidential Enquiry into Patient Outcome and Death for critical care rehabilitation, which collected data more recently, in 2022. The report identified significant failings in rehabilitation provision across the continuum of recovery, including during the post‐ICU in‐hospital period (National Confidential Enquiry into Patient Outcome and Death 2025). Therefore, it is likely that the findings of this study are still highly relevant to current post‐ICU populations, offering an opportunity to move closer to the vision of a critical care without walls.

### 6.5 Recommendations for Further Research

Future research could consider operational criteria to define wardability to further support discharge decision making and enhance patient safety.

### 6.6 Implications for Policy and Practice

The findings of this study demonstrate that by recognising the tension between being ready for ICU discharge (i.e., not requiring organ support) and being wardable (having care needs which can be fulfilled on the ward they are being discharged to), clinicians may be able to put in place support to ensure these high care needs are met. This has the potential not only to support high quality care resulting in better outcomes for post‐ICU patients, but also improve their experience by reducing the fear and anxiety experienced by them and the staff and family members caring for them.

## 7 Conclusion

The term *wardable* is used by staff to indicate the suitability or otherwise of patient transfer and has its counterpart in patients' and family members' experiences. This study identified a tension between being deemed wardable from an ICU perspective (no longer needing organ support) and being sufficiently recovered that continuing needs can be delivered within the current system of ward care in the NHS. This mismatch exacerbates the perception of otherness and compounds the fear and anxiety related to post‐ICU care experienced by both patients and the staff caring for them. Recognising this and putting support in place to manage the characteristics of otherness on the ward has the potential to both improve patient safety and patient and staff experience.

## Author Contributions

**Sarah Vollam:** conceptualisation; data curation; formal analysis; funding acquisition; investigation; methodology; project administration; writing – original draft preparation. **Melanie Gager:** formal analysis; investigation; project administration; writing – review and editing. **Hilary Thomas:** formal analysis; methodology; supervision; writing – review and editing. **Peter Watkinson:** conceptualisation; funding acquisition; resources; supervision; writing – review and editing. **Natalie Pattison:** formal analysis; methodology; supervision; writing – review and editing.

## Funding

This paper presents independent research funded by the National Institute for Health Research (NIHR) under its Research for Patient Benefit (RfPB) Programme (Grant Reference Number PB‐PG‐0215‐36149). This research was also supported by the National Institute for Health Research (NIHR) Oxford Biomedical Research Centre (BRC). The views expressed are those of the author(s) and not necessarily those of the NHS, the NIHR or the Department of Health.

## Ethics Statement

Ethical approval was granted in the UK by Wales REC 4 (reference 17/WA/0139) on 4th May 2017.

## Consent

All participants provided written informed consent prior to participation in this study.

## Conflicts of Interest

The authors declare no conflicts of interest.

## Data Availability

Data will be made available on request and with appropriate ethical approval.
